# Language-Agnostic Reproducible Data Analysis Using Literate Programming

**DOI:** 10.1371/journal.pone.0164023

**Published:** 2016-10-06

**Authors:** Boris Vassilev, Riku Louhimo, Elina Ikonen, Sampsa Hautaniemi

**Affiliations:** 1 Department of Anatomy, Faculty of Medicine, University of Helsinki, Helsinki, Finland; 2 Research Programs Unit, Genome-Scale Biology, University of Helsinki, Helsinki, Finland; 3 Minerva Foundation Institute for Medical Research, Helsinki, Finland; Swiss Institute of Bioinformatics, SWITZERLAND

## Abstract

A modern biomedical research project can easily contain hundreds of analysis steps and lack of reproducibility of the analyses has been recognized as a severe issue. While thorough documentation enables reproducibility, the number of analysis programs used can be so large that in reality reproducibility cannot be easily achieved. Literate programming is an approach to present computer programs to human readers. The code is rearranged to follow the logic of the program, and to explain that logic in a natural language. The code executed by the computer is extracted from the literate source code. As such, literate programming is an ideal formalism for systematizing analysis steps in biomedical research. We have developed the reproducible computing tool Lir (**li**terate, **r**eproducible computing) that allows a tool-agnostic approach to biomedical data analysis. We demonstrate the utility of Lir by applying it to a case study. Our aim was to investigate the role of endosomal trafficking regulators to the progression of breast cancer. In this analysis, a variety of tools were combined to interpret the available data: a relational database, standard command-line tools, and a statistical computing environment. The analysis revealed that the lipid transport related genes *LAPTM4B* and *NDRG1* are coamplified in breast cancer patients, and identified genes potentially cooperating with *LAPTM4B* in breast cancer progression. Our case study demonstrates that with Lir, an array of tools can be combined in the same data analysis to improve efficiency, reproducibility, and ease of understanding. Lir is an open-source software available at github.com/borisvassilev/lir.

## Introduction

The results of a study can be reproduced and evaluated when all data has been disclosed [1] and the computational methods have been shared in detail [2]. A study of 18 published data analyses showed that the majority of the analyses could not be reproduced, often due to the incomplete specification of the data processing and the analysis [3]. To improve reproducibility of computational analyses several guidelines have been suggested. For example, Sandve et al. proposed a list of ten simple rules for reproducible computational research [4]. Wilson et al. compiled an itemized list of best practices for scientific computing [5], and Shade et al. presented a step-by-step guide to computational analysis aimed at biologists [6]. While it would be beneficial to a data analyst to follow stringently all provided guidelines, there is a paucity of computer software that facilitates the implementation of all of them.

Generic software used in programming, such as version control and build utilities, cover some of the needs for reproducible analysis. Other software is specifically aimed at computational data analysis. One such software, Sweave, allows embedding R code into a document typeset with L^A^T_E_X [7]. The results of the automated data analysis described in the embedded code are inserted into the generated report to guarantee reproducibility. The utility of Sweave inspired an improvement, Knitr, that addresses most of the perceived shortcomings of its predecessor [8]. Both tools offer an electronic, automated version of the “lab notebook” as described by Noble [9] for the R Statistical Environment [10]. IPython is a notebook solution for the Python programming language [11]. It has evolved into Jupyter (jupyter.org), a platform which supports reproducible computing notebooks in many programming languages, and has become widely accepted [12]. A curated list of publications that employ such notebooks, with links to the data analyses, is available at go.nature.com/mqonbm.

Complex frameworks for the integration of heterogeneous, large-scale biological data have also been developed [13, 14]. An interesting solution proposed by Kitchin [15] addresses the problem of sharing the data analysis in journal publications by embedding the computer executable code within the published PDF.

Existing solutions either assume the exclusive use of a single programming language, such as R or Python [7, 8, 11], or require a non-trivial tool chain and a domain specific language [13, 14]. Here, we introduce Lir: a tool for reproducible computing that encourages and simplifies the use of any combination of existing software platforms and programming languages within the same data analysis [16].

Lir is based on the idea of literate programming as proposed by Donald Knuth [17]. Literate programming allows the user to organize and document their work free of the restrictions on code placement, file structure, or naming imposed by programming languages. This is achieved by embedding all code within named paragraphs called *code chunks*. Code chunks may appear in any order within the literate source file. In the original implementation by Knuth, a code chunk would immediately follow the text that explains the rationale of the code and its importance within the logic of the whole program; Lir follows that convention. Code chunks have descriptive names in natural language, and can contain other code chunks through name references. Thus, all computer code can be presented as the logic of the analysis requires. Additionally, Lir provides facilities for combining programs written in any programming language in the same data analysis. For instance, a relational database can be used to organize and query the data, a scientific computing platform to analyze it, and a statistical computing environment to summarize and visualize it.

The complete analysis—documentation, source code, placeholders for display items, discussion of results—is maintained as a text file. This file (S1 File) contains code chunks in the syntax recognized by the most widely used language-agnostic literate programming tool, noweb. This source file is used to dynamically generate all results and the final human-readable document (S2 File).

To demonstrate the application of Lir, we studied the effects of endosomal trafficking regulators on the progression of breast cancer by using gene amplification, mRNA expression, and patient survival data from The Cancer Genome Atlas repository [18]. Endosomal trafficking is the process by which cells internalize, sort, and recycle nutrients and signaling molecules with the help of vesicles formed at the cell’s outer membrane, the plasma membrane. Defects in endosomal uptake, sorting, recycling, and degradation of cell metabolites, external signals, and cell surface receptors can lead to deregulation of the cell cycle. The focus of the study was on genes and gene products involved in endosomal lipid trafficking and in breast cancer progression. The complete analysis is available as a supplement (S1 File).

## Methods

### Lir

**Fig 1:**
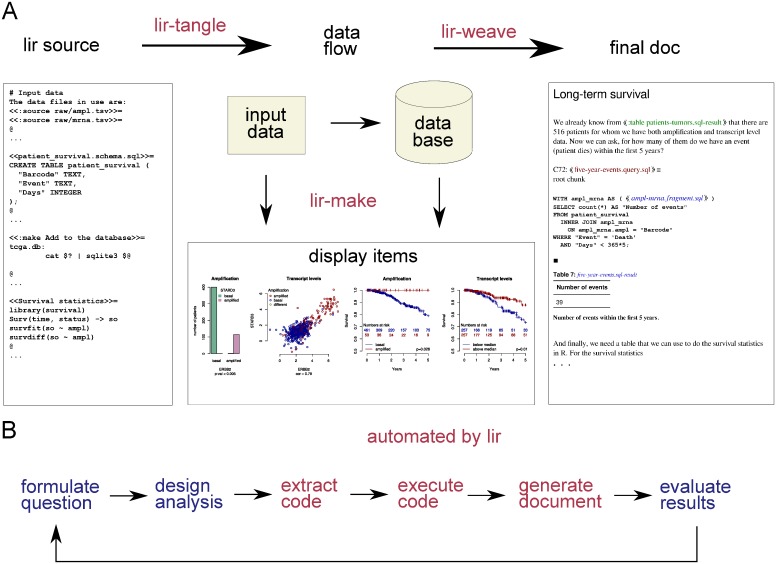
Overview of the method and tools provided by Lir. **A**: Documented executable code, placeholders for the display items, and discussion of the displayed results are maintained as a text file: the Lir source. Dependencies between the input data (data objects) and the executable code (data transformations) are an integral part of the source. The executable code and instructions for running it are extracted from the source file by lir-tangle. The code is evaluated by lir-make to obtain results and display items. A human-readable, cross-linked document that faithfully represents the literate source text file and integrates all display items is compiled by lir-weave. **B**: An overview of the work flow using this method. All steps that are performed by a computer are automated, including resolving dependencies between data objects and data transformations. This facilitates exploratory data analysis with short iteration cycles between formulating a question, evaluating its answer, and refining or extending the analysis.

Lir defines a markup language for defining and documenting a data analysis, and presenting its results. The data analysis is defined by the declared data objects (data files), the defined data transformations (executable programs that consume and produce data files), and the declared rules for applying a data transformation on data objects. Lir provides three command-line programs, lir-tangle, lir-make, and lir-weave, that generate the results of the data analysis and compile a human-readable document (Fig 1A). For most use cases, the three steps can be invoked in simple succession; the convenience program lir does that. The Tutorial that accompanies the Lir implementation gives an introduction to how Lir is meant to be used, and the User Guide provides a complete reference (available at https://github.com/borisvassilev/lir and https://github.com/borisvassilev/lir-tutorial).

#### Source file

A Lir source file is a valid noweb source file. The noweb syntax splits a text file into a sequence of documentation chunks and code chunks, and imposes no restrictions on the content of these chunks [19]. Code chunks are named, and names can be arbitrary strings, containing for example formatted text or mathematical symbols and formulas. Code chunks can include other code chunks through name references. Code chunks that are not referenced by other code chunks are *root* code chunks. The executable programs evaluated during the data analysis are defined in root code chunks named after the transformation object they represent. Documentation chunks can be formatted and structured using any markdown recognized by Pandoc.

#### Analysis definition

Lir extends the semantics of noweb by embedding in code chunks with special names the dependencies between executable code, input data, and results. The data objects, data transformations, and the rules describe a directed acyclic graph (DAG): the data objects are the vertices of the graph, and the rules for applying data transformations are the directed edges. In this DAG, the sources are the input data files; the final results and figures are the sinks. The input data files (the sources) must be explicitly declared. If a placeholder for a display item (for example, a table or a figure) appears in the Lir source file, the file containing the display item is a sink. Additional sinks can be declared for results that are not displayed in the final document but have to be generated. The rules for applying a data transformation on data objects are declared in their own code chunks, in the language recognized by Make. In these rules, data objects are referred to by the names used in the declarations of sources and sinks. Data transformations are referred to by the root code chunk names in which they are defined.

#### Generating results

In a first step, lir-tangle extracts the data transformations defined in the source file to executable programs, and uses the declared data objects and rules to build a valid makefile. In the next step, lir-make invokes Make to generate all results, observing the dependencies between input data, intermediate files, and final results. By keeping this step separate it is possible to execute it on a different machine, for example a remote application server. Only the tools used in the data analysis and Make have to be installed on the machine running the data analysis: neither Lir, nor any of its dependencies (noweb, SWI-Prolog, Pandoc, Bash, and so on) are required.

#### Compiling the final document

In the last step, lir-weave produces an HTML document that contains the full text of the source file, all display items, and links to all generated files that are not displayed (see S2 File). The code chunks containing the executable programs are numbered and cross-linked to help browsing and reading the code. If markdown is used in the documentation chunks, the final document is formatted accordingly and has a table of contents. Figures, tables, and plain text results are displayed with their own labels (“Figure”, “Table”, “Listing”), may have captions, and are numbered separately.

#### Implementation

Tangling the source file with lir-tangle is implemented as a Bash script that makes use of the low-level tools provided by noweb, and standard Linux command line tools like Awk, sed, and grep. Generating all results with lir-make is implemented as an invocation of Make with a makefile generated by lir-tangle and the appropriate command-line options. The implementation of lir-weave employs two steps. First, the lir source file is converted to an intermediate representation. This is implemented in SWI-Prolog [20], as it is a convenient tool for both parsing the Lir source file and generating HTML markup. In this step, code chunks and display items are numbered, cross-linked, and structured using HTML span and div elements modified with HTML attributes. The intermediate representation is compiled to a final document using Pandoc and formatted with a default CSS style sheet provided by Lir. Compiling the final document with lir-weave may be extended to produce other output formats, for example a PDF file for printing. We chose HTML as the first supported final document format for two reasons. First, it is a format that can be viewed any platform with a modern web browser. Second, the final layout and formatting can be customized by the user by providing another CSS style sheet, without changing or extending the implementation.

#### Compatibility

The tools provided by Lir and all its dependencies are open source software that can be installed on any GNU/Linux, BSD, and OS X. It is possible to install and use Lir on a Microsoft Windows system, but the differences in file path naming conventions between POSIX and Microsoft Windows might cause incompatibilities at the level of the Lir source file. We cannot currently guarantee, for example, that a Lir source file that contains file paths with backslashes as component separators or file names with spaces (allowed by the Microsoft Windows Uniform Naming Convention) can be interpreted on a POSIX system without normalizing the file paths.

### Breast cancer data

The raw data on gene amplification and mRNA levels for breast cancer patients were obtained from The Cancer Genome Atlas project (TCGA) [18] and pre-processed to obtain two data matrices: the amplification status and mRNA levels for approximately 18 thousand genes for tissue samples from over 500 patients.

We downloaded level 1 Affymetrix SNP Array data from the Cancer Genome Atlas [18]. The data was processed anonymously. All TCGA data were preprocessed using Anduril [13]. We genotyped the probes, and estimated and normalized copy-number values to 2 with the CRLMM algorithm [21]. Copy-number data were segmented with the circular binary segmentation (CBS) algorithm using the R package DNAcopy (parameters undo.splits = sdundo, SD = 3, alpha = 0.01) [22]. Copy-numbers for each gene were assigned to three states (gained, normal and deleted) similarly to TCGA [18].

Gene expression had been measured with an Agilent two-channel microarray from which only the channel containing measurements from a patient sample were used. We mapped probes to genes, and removed probes mapping to multiple genes or no genes. Gene expression values for genes, which are tagged by several probes, were combined using the median over these probes. Gene expression values were normalized to a mean of 0 over the samples.

For each gene and sample, the amplification value was either 0 (no amplification) or 1 (gene is amplified). The mRNA level was represented as a numerical value that can be compared between patient samples for the same gene. Each gene was uniquely identified by an Ensembl Gene ID. Each sample was uniquely identified by a TCGA “barcode” that contains meta-information including, among others, details about the collection site, sample type, and study participant. Clinical data for all patients, including survival data, was also obtained from TCGA.

### Statistical methods and visualizations

The overall goal of the case study was to see how well the genes that regulate endosomal trafficking correlate at mRNA, miRNA and copy-number levels. In the copy-number correlation analyses we used the *χ*^2^ test statistic for a 2 × 2 contingency table that represents the amplification status values for each pair of genes. The contingency table itself was visualized as a bar plot where each bar represents one of the table cells. The correlation of the mRNA levels was estimated using the Pearson’s correlation coefficient. All plots were generated using R’s built-in methods except for survival curves, which were generated using R’s survival package [23, 24].

## Results and Discussion

In order to demonstrate Lir, we analyzed breast cancer data including amplification status, mRNA level data, and survival data from 516 patients. The analysis employed a relational database, an array of standard command-line tools for text and table manipulation, and the R statistical environment for statistical analysis and visualization. The results were obtained using the basic work flow outlined in Fig 1B.

We were initially interested in three genes. These genes code for proteins implicated both in endosomal lipid trafficking and in breast cancer. StARD3 (**StAR**-related lipid transfer **d**omain protein **3**) is a cholesterol-binding late endosomal (LE) protein that contributes to the progression of ErbB2-positive breast cancer, an established aggressive cancer sub-type [25]. NDRG1 (**N**-myc **d**ownstream **r**egulated **g**ene **1**), implicated in several cancers [26], regulates endosomal trafficking and degradation of the cell-surface receptor for low-density lipoprotein [27]. LAPTM4B (**L**ysosomal-**a**ssociated **t**rans**m**embrane **p**rotein **4**-**b**eta) is a LE membrane protein associated with chemotherapy resistance in several cancers. Recent results show that it controls ceramide export from the LE and thereby affects sensitivity to anti-cancer drugs [28]. Altered *STARD3*, *NDRG1*, or *LAPTM4B* expression has been associated with neoplasms in multiple reports. According to experimental findings in cell culture models, all three proteins function at different points along the endosomal trafficking route, and regulate intracellular lipid trafficking. It was therefore of interest to assess if their gene amplification or mRNA expression levels correlate with each other or with breast cancer patient survival.

### Correlating genes of interest

**Fig 2:**
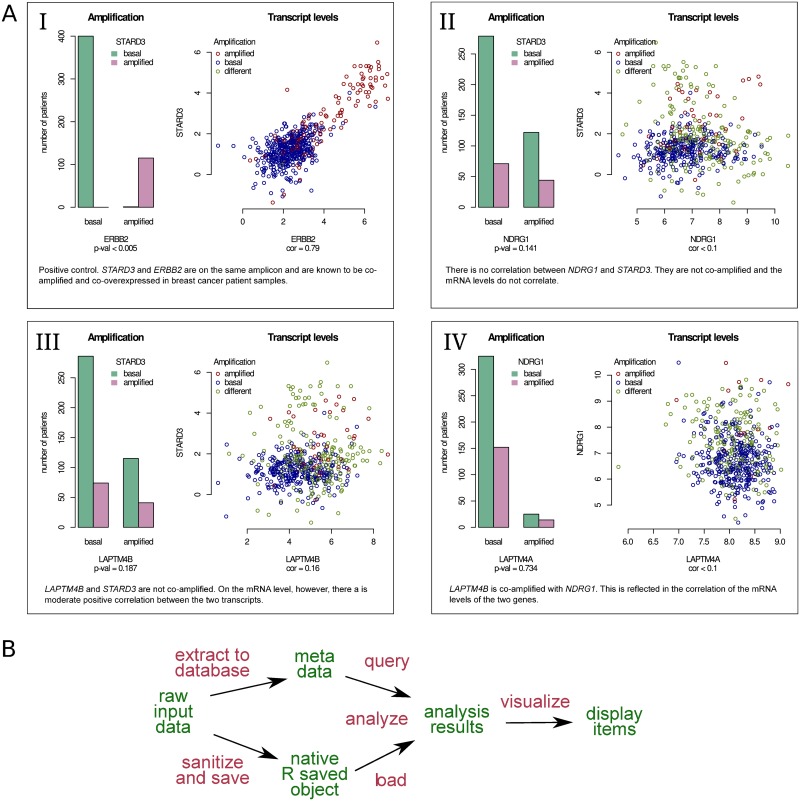
Using Lir to correlate genes of interest. **A**: Four of the display items generated by the analysis outlined in (A). In the scatter plots, red indicates a sample in which both genes were amplified, blue indicates a sample in which both genes are at basal levels, and green indicates a sample with differential amplification status of the two genes. The interpretation of the results of each display item shown here is added to the source file: the interpretation becomes an integral part of the analysis. **B**: Diagram demonstrating the data flow for an analysis. The input data (a large data matrix in a text file) is sanitized and saved as a native R object. Meta-data of the genes and the samples is saved to a relational database to facilitate querying the data. The relevant data is extracted, analyzed, and visualized, producing several display items. In this diagram, data objects are colored in green, data transformations are colored in red, and arrows represent the dependencies declared in the source file and used by Lir to generate the intermediate data objects and the display items.

The genes of interest, *STARD3*, *NDRG1*, and *LAPTM4B* are located in the following chromosomal regions: *STARD3* in the *ERBB2* amplicon in 17q12-21 [29], *NDRG1* in 8q12-24, often coamplified with MYC [30], and *LAPTM4B* on the same chromosome arm in 8q22 [31], approximately 35 Mb apart from the *NDRG1* locus. First, we investigated whether the gene amplification or mRNA levels of *STARD3*, *NDRG1*, and *LAPTM4B* correlate with each other in breast cancer tumor tissue. As a positive control we calculated correlation between *STARD3* and *ERBB2*, which are known to be highly correlated on the DNA, mRNA, and protein level [25, 29, 32]. The amplification statuses of these two genes is almost identical, and they correlate strongly at the mRNA level (Pearson’s *ρ* = 0.79, Fig 2A, Panel I).

We found no correlation between *NDRG1* and *STARD3* amplification status or mRNA levels (Pearson’s *ρ* < 0.1, Fig 2A, Panel II). Instead, a weak correlation between *STARD3* and *LAPTM4B* was observed. Although the amplification status of these two genes did not correlate, on the mRNA level there was a weak positive correlation (Pearson’s *ρ* = 0.16, Fig 2A, Panel III). This suggests that the two genes might be co-regulated on the transcriptional or post-transcriptional level. Interestingly, *LAPTM4B* and *NDRG1* correlate positively both in their gene amplification status and mRNA levels (Pearson’s *ρ* = 0.34, Fig 2A, Panel IV). This may be related to their close proximity in the 8q region amplified in cancers.

An overview of the data flow used to generate the above results is outlined in Fig 2B. A range of tools are used: standard command line tools for examining and preparing the input data (wc, sed, awk, tr, cut, etc.), a relational data base (SQLite) for the meta data, R for the statistical analysis and plot generation. Code chunks are given descriptive names in free text, allowing for a self-documenting, consistent, literate writing style independent of the programming language used (for example, see S1 File, lines 618–702). Scripting tools like R offer little flexibility when it comes to positioning code within the file or sharing code between files. Multiple references by name to the same code chunk can be used to avoid code repetition and reorganize code, overcoming this practical limitation (see S1 File, line 264, used at 225, 535, 598, 894, …).

### Analyzing the effect of genes of interest on patient survival

We next assessed whether the overexpression of a gene or a pair of correlated genes have an effect on patient survival within five years. We have earlier reported that high StARD3 protein levels associate with poor breast cancer specific survival in two Finnish nationwide patient cohorts [25]. This was, however, not observed on the amplification or transcript level in the TCGA data set (S2 File). Thus, we tested the survival effect of *SERPINA1*, another recently reported predictor of survival in breast cancer [33].

**Fig 3:**
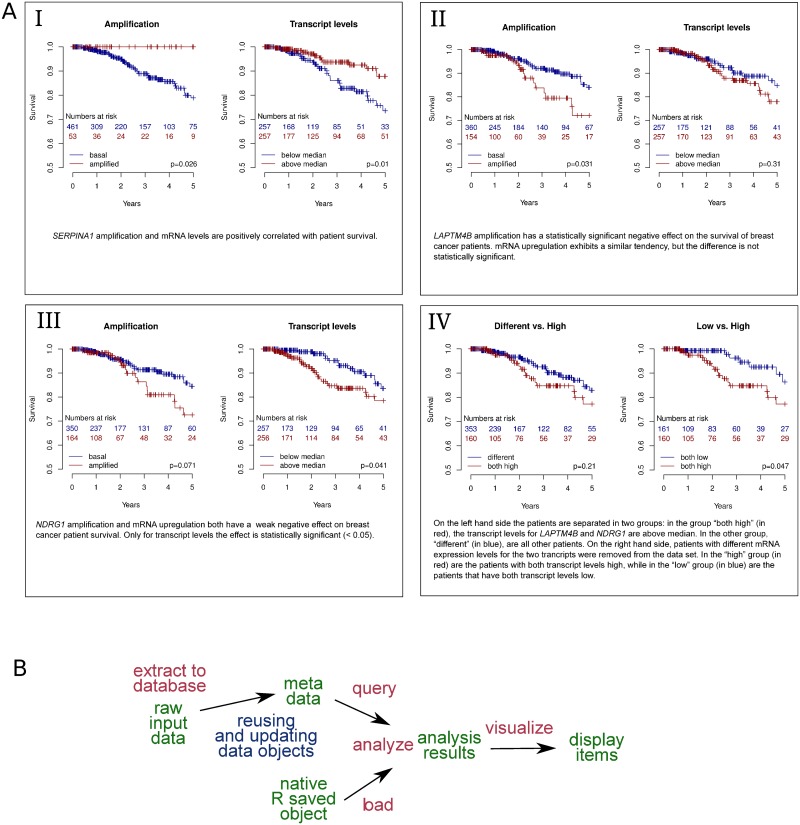
Using Lir to determine the effects of upregulation of the genes of interest on patient survival. **A**: Three of the display items generated by the analysis outlined in (A). As in Fig 2, the interpretations of the results shown below each display item appear verbatim in the literate source. **B**: Diagram demonstrating the data flow for further analysis based on the results shown in Fig 2. Importantly, data objects generated in the previous analysis are reused. In this diagram, data objects are colored in green, data transformations are colored in red, and arrows represent the dependencies declared in the literate source file and used by Lir to generate the intermediate data objects and the display items.

A clear positive correlation of both *SERPINA1* amplification status and mRNA levels with patient survival was observed (Fig 3A, Panel I). On the other hand, both *LAPTM4B* and *NDRG1* levels correlated with reduced patient survival: in the case of *LAPTM4B*, the negative effect was evident at the level of gene amplification (Fig 3A, Panel II), while for *NDRG1*, the correlation was better at the transcript level (Fig 3A, Panel III).

To test the combined effect of *LAPTM4B* and *NDRG1* mRNA overexpression on patient survival, the patients were split into two groups. First, one group contained those patients that have high mRNA levels (over median within the gene) for both genes, and the other group all other patients (Fig 3A, Panel IV). These groups had similar survival. Then, the mixed mRNA level patients (one mRNA below, the other above median) were excluded from the data set and only those patients with high or low mRNA levels for both transcripts were compared with each other. This revealed a lower survival for patients with elevated transcript levels; however, the effect was similar to that obtained by *NDRG1* upregulation alone, implying that co-overexpression of *LAPTM4B* and *NDRG1* did not have an additional negative effect on patient survival in this data set.

The data flow of these analyses is summarized in Fig 3B. The data flow documents how the reuse of existing data objects avoids the need for excess code and lowers the amount of computational work. The structure of the data flow strongly resembles that of the work flow presented in Fig 2B. The formal syntax used by Lir for declaring dependencies allows to reuse existing data flow structures, further avoiding unnecessary work (see S1 File, lines 798–801, reusing lines 285–90, 293–7, and 301–5).

### Assessing miRNA target genes by integrating two independent data sets

**Fig 4:**
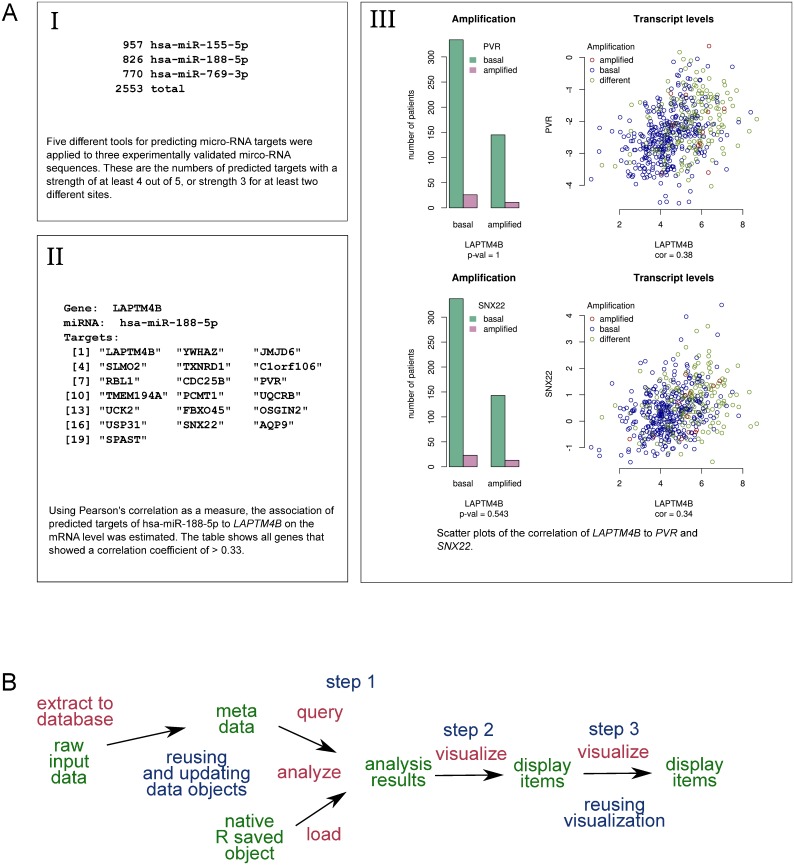
Using Lir to integrate two independent data sets and visualize the results. **A**: The display items generated by the analysis outlined in (B). The result of each step, represented by the corresponding display item, is taken into consideration when formulating the next question and designing the analysis. **B**: Diagram demonstrating the data flow for an analysis that incorporates a new data set. The new data set associates genes with a measure of the certainty that they are targeted by the same micro-RNA as a gene of interest. The existing relational database object was updated (not re-generated) to include the additional data. The analysis was done in three consecutive steps: First, a reasonable cut-off for the prediction certainty of the micro-RNA targets was determined (Step 1); then the genes associated to the gene of interest were found (Step 2); then, the mRNA levels of the most interesting of the associated genes was plotted against the mRNA levels of the gene of interest (Step 3). The visualization from Fig 2B was reused in the last step. Data objects are colored in green, data transformations are colored in red, and arrows represent the dependencies.

Micro RNA (miRNA) are short non-coding RNA molecules that regulate mRNAs after transcription, usually inducing gene silencing [34, 35]. Individual miRNAs may target as many as 100 different mRNA molecules. For three of the genes of interest, we identified experimentally validated miRNAs relevant in the context of breast cancer: for *ERBB2*, miR-155 [36], for *LAPTM4B*, miR-188 [37], and for *NDRG1*, miR-769 [38]. To find potential additional targets for each miRNA, we used miRWalk [39], additionally querying four more online resources: microRNA.org [40], miRDB [41], RNA22 [42], and TargetScanHuman [43]. There were 2553 genes in the TCGA breast cancer data set that were predicted targets with the selected cut-off (Fig 4A, Panel I). Of all the predicted targets that correlated with the corresponding gene on the transcript level (S2 File and Fig 4A, Panel II), two of the *LAPTM4B*/miR-188 genes were especially interesting: *PVR* (Poliovirus receptor protein), and *SNX22* (Sorting nexin-22). The Poliovirus receptor protein might provide tumors with a mechanism of immunoevasion, and it plays a role in mediating tumor cell invasion and migration [44]. Sorting nexin-22 may be involved in several stages of intracellular trafficking, inferred from sequence similarity, and contains binding sites for phosphatidylinositol 3-phosphate [45].

The contingency tables of the amplification status and the scatter plots of the mRNA levels of *LAPTM4B* and the two genes are shown in Fig 4A, Panel III. They allow for the following observations: the genes are clearly correlated on the mRNA level, despite the fact that they are not co-amplified. Thus, our data suggest that the mRNA levels of these genes are regulated by miRNA-188, together with *LAPTM4B* mRNA. The phenomenon of one miRNA targeting a complex of functionally related proteins is known [46, 47]. It is therefore possible that *LAPTM4B*, *PVR*, and *SNX22* contribute to shared functions.

Fig 4B outlines the data flow of the combined analyses performed. Existing intermediate results are not regenerated. For example, only the miRNA target data is inserted into the existing relational database, while the already present amplification and mRNA data object is used as it is. This minimizes the time for generating and visualizing new results, thus encouraging an iterative, exploratory approach to data analysis without sacrificing repeatability. In addition, code chunks support ad hoc reuse of code (S1 File, lines 1357–78). In most common use cases achieving code reuse with R’s package system is more complex and time-consuming. The use of Lir does not prevent us from using R’s package system or the corresponding code reuse paradigm of any other programming platform. Rather, Lir facilitates a systematic approach to organizing computer code that is independent of any particular tool and can be used only if deemed beneficial.

## Conclusions

In this study, we have introduced a tool for reproducible computing called Lir. We used Lir to analyze a heterogeneous data set in order to see whether there is putative coregulation between a set of endosomal trafficking regulators. In the analysis, we combined a relational database, an array of data manipulation tools, and a statistical analysis environment. Our results revealed a coamplification of the cancer and lipid transport related genes *NDRG1* and *LAPTM4B*, as well as new genes potentially co-regulated and cooperating with *LAPTM4B*.

The major contribution of Lir within the context of reproducibility is to demonstrate that it is possible and very advantageous to fully document and automate a work flow utilizing a combination of software tools. Using the best tool for each task reduces the total amount of code, thus reducing the opportunity for mistakes, and the amount of invested time [48, 49]. All results and the computer code that generated them are presented as a human-readable document. This document serves two equally important purposes during the development. First, it organizes and presents the intermediate results: the data analysis can be conducted in an iterative, exploratory fashion while faithfully documenting all steps. Second, it organizes, documents and presents all computer executable code: other scientists can inspect the analysis and verify the results. In combination with a version control system, Lir directly facilitates the implementation of best-practice guidelines as delineated in [4–6].

## Supporting Information

S1 File The complete data analysis. This is a plain text file that uses extended markdown as understood by Pandoc. The file is a valid noweb source file. It is available at github.com/borisvassilev/endobrca.
(LIR)

S2 File The generated human-readable document. The final document is an HTML web page that can be viewed with a web browser. It is a faithful representation of the literate source file, which has additionally been cross-linked and prettified. It is available at github.com/borisvassilev/endobrca.
(HTML)
