# Supplementary material for: Language-Agnostic Reproducible Data Analysis Using Literate Programming
Source: PLoS One. 2016 Oct 6;11(10):e0164023. doi: 10.1371/journal.pone.0164023 (PMC5053501; doi:10.1371/journal.pone.0164023)
Supplement: S2 File — The final document is an HTML web page that can be viewed with a web browser. It is a faithful representation of the literate source file, which has additionally been cross-linked and prettified. It is available at github.com/borisvassilev/endobrca. (HTML) [file pone.0164023.s002.html]

TCGA breast cancer data analysis


# TCGA breast cancer data analysis

## Boris Vassilev; Elina Ikonen

### 2016-05-27 13:39:36 UTC


- Input data
  - Amplification and transcript levels
  - Mapping gene IDs to names
  - Meta data: TCGA, Ensembl
- Gene association
  - Pairs of genes
  - Association results
  - Plotting the results
- Survival
  - Clinical patient data
  - Long-term survival
  - Effects on long-term patient survival
- Protein complexes regulated by micro-RNA
  - Micro-RNAs and predicted targets

# Input data

For all breast cancer patients, we have genome-wide amplification and transcript level data. Patients and samples are identified by their TCGA “barcodes”. There is relevant meta-data embedded in these barcodes.

Genes are identified by their Enseml Gene IDs.

## Amplification and transcript levels

Our collaborator Riku Louhimo has pre-processed the genome-wide data available from TCGA for all breast cancer samples and provided two sets of data. Both sets have one gene on every row, with each field representing one sample.

The first set contains DNA *amplification calls*: a matrix of 1 (Amplified), 0 (Not amplified), or `NA` (Not available). The other set contains mRNA *transcript levels*, a matrix of floating-point values or `NA` (Not available).

S1: `raw/ampl.tsv`

S2: `raw/mrna.tsv`

Both files are quite large, as seen in the ⟪ :listing rawdata.size ⟫.

Here is what Riku had to say about the data:

> Copy numbers are measured with Affy 6.0 SNP Arrays. Probes are genotyped, copy-number values are estimated and normalized to 2 with CRLMM. Copy-number data are segmented with the circular binary segmentation (CBS) algorithm using the R package DNAcopy (parameters `undo.splits=sdundo`, `SD=3`, `alpha=0.01`). Copy-number calls (1: amplification, 0: normal, −1: deletion) are made similarly to TCGA: in short, by assigning 1 to CNA regions with segment mean >2.3 and −1 to regions with segment mean <1.7.

> In the copy-number-call-matrix you have, `NA` (not available) values occur in genes for which copy-number is normal, unnchanged, or diploid in all samples. In the gene expression matrix, Agilent arrays contain control fields which denote several types of array or scanner errors and quality measures. `NA`-values in this matrix are therefore a result of the Agilent scanner giving a bad quality flag to a probe which we than convert to `NA`.

A couple of things to note:

- There are no −1 values in the “copy number calls” (amplification) matrix, and Riku has told me that I shouldn’t worry about it;
- The `NA` values in the amplification matrix should be zeroes (no amplification), at least when interpreting them in a biological context (this is handled in ⟪ Load the amplification matrix ⟫);
- When working with the mRNA (transcript level) matrix, we just remove any `NA` values – for example, in R, to get the median, use `median(mrna, na.rm=TRUE)`.

For both files, the rows are labelled with Ensembl IDs for each gene and the columns are labelled with TCGA barcodes representing patients or samples. The results of the query:

Listing 1: `rawdata.size`

```
21M	raw/ampl.tsv
187M	raw/mrna.tsv
207M	total
```

Raw data file sizes. The sizes of each of the raw data files, in a human-readable format.

C1: ⟪ :make ⟫ ≡  
 root chunk  
 definition continued in ↓ C2 + ⇣ C15 + ⇣ C16 + ⇣ C19 + ⇣ C22 + ⇣ C47 + ⇣ C60 + ⇣ C61 + ⇣ C62 + ⇣ C63 + ⇣ C64 + ⇣ C75 + ⇣ C77 + ⇣ C82 + ⇣ C87 + ⇣ C89 + ⇣ C90 + ⇣ C97

```
rawdata.size: raw/ampl.tsv raw/mrna.tsv
	du --dereference --human-readable --total $^ > $@
```

⇩ C2

Since both files are quite big, and saved in a plain text, it is prohibitively expensive to load them. To avoid this, we load them once from R and save the data as a native R object that is both smaller and much faster to load.

Listing 2: `input-data.Rsave.size`

```
82M	input-data.Rsave
```

Size of the native R data object. Once a tab-separated plain-text data matrix has been loaded and saved as a native R object, it becomes much smaller.

C2: ⟪ :make ⟫ ≡  
 ⇧ C1

```
input-data.Rsave.size: input-data.Rsave
	du --human-readable $^ > $@
```

⇩ C15

To keep the intermediate data object, we declare it as `SECONDARY` in the Makefile.

C3: ⟪ :make The R saved state ⟫ ≡  
 root chunk

```
.SECONDARY: input-data.Rsave
input-data.Rsave: input-data.R \
                  ampl.rownames \
                  ampl.colnames \
                  ampl.vals \
                  mrna.rownames \
                  mrna.colnames \
                  mrna.vals
	Rscript --vanilla $^ $@
```

◼

The script that reads the plain text data matrices and saves them to native R objects. It takes the names of all files as command line arguments.

C4: ⟪ input-data.R ⟫ ≡  
 root chunk

```
args <- commandArgs(trailingOnly = TRUE)
names(args) <- c("ampl.rownames",
                 "ampl.colnames",
                 "ampl.vals",
                 "mrna.rownames",
                 "mrna.colnames",
                 "mrna.vals",
                 "save.file")

⟪ Load the amplification matrix ⟫
⟪ Load the mRNA matrix ⟫

save(ampl, mrna, file=args["save.file"])
```

◼

Here, the second line makes all `NA` values 0 (no amplification). The third one makes the amplification a truth value: `FALSE` for basal level and `TRUE` for amplification of the gene.

C5: ⟪ Load the amplification matrix ⟫ ≡  
 Appears in C4

```
ampl <- scan(file=args["ampl.vals"], what=integer())
ampl[is.na(ampl)] <- 0
ampl <- as.logical(ampl)
ampl.rownames <- scan(file=args["ampl.rownames"], what=character())
ampl.colnames <- scan(file=args["ampl.colnames"], what=character())

ampl <- matrix(ampl,
               nrow=length(ampl.rownames), ncol=length(ampl.colnames),
               dimnames=list(ampl.rownames, ampl.colnames),
               byrow=TRUE)
```

◼

C6: ⟪ Load the mRNA matrix ⟫ ≡  
 Appears in C4

```
mrna <- scan(file=args["mrna.vals"], what=numeric())
mrna.rownames <- scan(file=args["mrna.rownames"], what=character())
mrna.colnames <- scan(file=args["mrna.colnames"], what=character())

mrna <- matrix(mrna,
               nrow=length(mrna.rownames), ncol=length(mrna.colnames),
               dimnames=list(mrna.rownames, mrna.colnames),
               byrow=TRUE)
```

◼

C7: ⟪ :make Matrix values and row and column labels ⟫ ≡  
 root chunk

```
%.vals: get-vals.bash raw/%.tsv
	bash $^ > $@

%.rownames: get-rownames.bash raw/%.tsv
	bash $^ > $@

%.colnames: get-colnames.bash raw/%.tsv
	bash $^ > $@
```

◼

C8: ⟪ get-vals.bash ⟫ ≡  
 root chunk

```
< "$1" ⟪ Drop first row ⟫ | ⟪ Drop first column ⟫
```

◼

C9: ⟪ get-rownames.bash ⟫ ≡  
 root chunk

```
< "$1" ⟪ Drop first row ⟫ | ⟪ Get first column ⟫
```

◼

C10: ⟪ get-colnames.bash ⟫ ≡  
 root chunk

```
< "$1" ⟪ Get first row ⟫ | ⟪ Drop first column ⟫
```

◼

C11: ⟪ Drop first row ⟫ ≡  
 Appears in C8, C9, C62, C63, C65, C89

```
sed -n '2~1p'
```

◼

C12: ⟪ Get first row ⟫ ≡  
 Appears in C10, C60, C61, C87

```
sed '1q'
```

◼

C13: ⟪ Drop first column ⟫ ≡  
 Appears in C8, C10, C65

```
cut --complement --fields=1
```

◼

C14: ⟪ Get first column ⟫ ≡  
 Appears in C9, C15

```
cut --fields=1
```

◼

## Mapping gene IDs to names

All genes are identified by their Ensembl IDs. It would be convenient to be able to select genes based on the canonical names of their protein products. To do that, we donwloaded a table that maps Ensembl gene IDs to a short description of the gene and the associated gene name. This was done using the “Customize your download” feature on the Ensembl downloads page, with the following settings:

Database: “Ensembl Genes 77”;

Dataset: “Homo sapiens genes (GRCh38)”;

Filters: “with HGNC ID(s): Only”;

Attributes: “Ensembl Gene ID”, “Description”, and “Associated Gene Name”.

The results of the query were downloaded as a tab-separated (TSV) file with a header and saved as:

S3: `ensembl/id_descr_name.tsv`

Already we can check if any of the gene IDs in either of the two data matrices is **not** in this list.

Listing 3: `mystery-counts`

```
 1729 mystery-genes-ampl
 1815 mystery-genes-mrna
 3544 total
```

Number of genes without names. There are quite a few genes without names; about 10% of the total.

C15: ⟪ :make ⟫ ≡  
 ⇧ C2

```
mystery-counts: mystery-genes-ampl mystery-genes-mrna
	wc --lines $^ > $@

mystery-genes-%: sorted-genes-% sorted-genes-known
	comm -2 -3 $^ > $@

sorted-genes-%: %.rownames
	sort --unique $^ > $@

sorted-genes-known: ensembl/id_descr_name.tsv
	⟪ Get first column ⟫ $< | sort --unique > $@
```

⇩ C16

We can already do a sanity check of the data. We will compare two genes that we know should be co-amplified in almost all samples, and correlate very well on the transcript level: *ERBB2* and *STARD3*. We would have to find their IDs from the file `ensembl/id_descr_name.tsv`, and use these IDs to pick out the amplification and mRNA data from the saved R data object `input-data.Rsave`. This would be an informal validation of all work so far.

Figure 1: `sanity-check.svg`

Sanity check. As expected, *STARD3* and *ERBB2* are almost always co-amplified and their transcript levels correlate very strongly.

C16: ⟪ :make ⟫ ≡  
 ⇧ C15

```
sanity-check.ids: sanity-check.bash ensembl/id_descr_name.tsv
	bash $^ > $@
```

⇩ C19

C17: ⟪ sanity-check.bash ⟫ ≡  
 root chunk

```
awk -v name='^STARD3$' '⟪ ID from name .awk ⟫' "$1"
awk -v name='^ERBB2$' '⟪ ID from name .awk ⟫' "$1"
```

◼

C18: ⟪ ID from name .awk ⟫ ≡  
 Appears in C17

```
BEGIN { FS = "\t"; OFS = "\t" }
$3 ~ name {
    print $3, $1
    exit
}
```

◼

C19: ⟪ :make ⟫ ≡  
 ⇧ C16

```
sanity-check.svg: sanity-check.R input-data.Rsave sanity-check.ids
	Rscript --vanilla $^ $@
```

⇩ C22

C20: ⟪ sanity-check.R ⟫ ≡  
 root chunk

```
args.names <- c("save.file", "ids.file", "fig.file")
⟪ Read and name R command line arguments ⟫

ids.table <- read.delim(args["ids.file"], header=F, row.names=1)
ensembl.id <- as.character(ids.table[,1])
names(ensembl.id) <- rownames(ids.table)

load(args["save.file"])

ampl[ensembl.id["STARD3"],] -> stard3.ampl
mrna[ensembl.id["STARD3"],] -> stard3.mrna
ampl[ensembl.id["ERBB2"],] -> erbb2.ampl
mrna[ensembl.id["ERBB2"],] -> erbb2.mrna

chisq.test(erbb2.ampl, stard3.ampl) -> chisq.test.result

svg(filename=args["fig.file"], width=7, height=4.7, pointsize=11)
layout(matrix(c(1,2), nrow=1, ncol=2, byrow=T),
       widths=c(2,3),
       heights=c(1,1))

# amplification
ampl.c <- hcl(c(140,320), l=80)
barplot(t(chisq.test.result$observed), beside=T, col=ampl.c,
        xlab="ERBB2 ampl",
        ylab="number of patients")
legend("topright",
       title="STARD3 ampl",
       legend=c("FALSE", "TRUE"),
       fill=ampl.c, border=ampl.c,
       bty="n",
       cex=.9)

# mRNA
plot(erbb2.mrna, stard3.mrna,
     xlab="ERBB2 mRNA level", ylab="STARD3 mRNA level",
     bty="n")

dev.off() -> foo
```

◼

C21: ⟪ Read and name R command line arguments ⟫ ≡  
 Appears in C20, C49, C52, C76, C78, C83, C94, C98, C100

```
args <- commandArgs(trailingOnly=T)
names(args) <- args.names
```

◼

## Meta data: TCGA, Ensembl

We will use a relational database to keep and query the TCGA meta data.

Listing 4: `tcga.db.schema`

```
CREATE TABLE patient_survival (
  "Barcode" TEXT,
  "Event" TEXT,
  "Days" INTEGER
);
CREATE TABLE mirna (
  mirna TEXT,
  gene TEXT,
  refseqid TEXT,
  sum INTEGER
);
CREATE TABLE ampl_rownames(
  "Ensembl Gene ID" TEXT
);
CREATE TABLE mrna_rownames(
  "Ensembl Gene ID" TEXT
);
CREATE TABLE portion_analyte(
  "Code" TEXT,
  "Definition" TEXT
);
CREATE TABLE sample_type(
  "Code" TEXT,
  "Definition" TEXT,
  "Short Letter Code" TEXT
);
CREATE TABLE tissue_source_site(
  "TSS Code" TEXT,
  "Source Site" TEXT,
  "Study Name" TEXT,
  "BCR" TEXT
);
CREATE TABLE id_descr_name(
  "Ensembl Gene ID" TEXT,
  "Description" TEXT,
  "Associated Gene Name" TEXT
);
CREATE TABLE ampl_cols(
  "Barcode" TEXT,
  "TSS Code" TEXT,
  "Patient" TEXT
);
CREATE TABLE mrna_cols(
  "Barcode" TEXT,
  "TSS Code" TEXT,
  "Patient" TEXT,
  "Sample" TEXT,
  "Vial" TEXT,
  "Portion" TEXT,
  "Analyte" TEXT
);
```

The complete schema so far.

This is the complete schema for the database, generated automatically from the database file.

C22: ⟪ :make ⟫ ≡  
 ⇧ C19

```
tcga.db.schema: tcga.db
	echo '.schema' | sqlite3 $< > $@
```

⇩ C47

To insert tables in to the database, we directly import tab-separated files with headers to a table named as the file, with the `.tsv` extension stripped. This uses a useful command of the SQLite shell, `.import`. When the table with that name has not been defined yet, the header line in the imported file is used.

C23: ⟪ create-table.bash ⟫ ≡  
 root chunk

```
NAME=$(basename --suffix=.tsv "$1")
echo 'DROP TABLE IF EXISTS "'"$NAME"'";'
echo '.separator "\t"'
echo ".import '$1' $NAME"
```

◼

This defines an implicit rule that generates the SQL code for inserting a table, using ⟪ create-table.bash ⟫ defined above, if the appropriate `.tsv` file exists.

C24: ⟪ :make Implicit rule for SQL tables from files with headers ⟫ ≡  
 root chunk

```
%.create-table.sql: create-table.bash %.tsv
	bash $^ > $@
```

◼

This is the rule that builds and updates the database. Note that only changes will be applied to the database.

C25: ⟪ :make Populate the relational database ⟫ ≡  
 root chunk

```
tcga.db:
	cat $? | sqlite3 $@
```

◼

In the amplification data matrix, column labels denote patients. In the transcript level matrix, column labels denote samples. Both patients and samples are labeled by a TCGA barcode. Those barcodes contain meta-information: for example, using the barcode, we can separate solid tumor samples from healthy tissue control samples in the transcript level data matrix.

The different identifiers within a barcode are described in “code table reports” available for download. The following three code table reports are relevant for us:

S4: `tcga/portion_analyte.tsv`

S5: `tcga/sample_type.tsv`

S6: `tcga/tissue_source_site.tsv`

C26: ⟪ :make Code table reports to the database ⟫ ≡  
 root chunk

```
tcgatables := $(patsubst %.tsv,%.create-table.sql,$(wildcard tcga/*.tsv))
tcga.db: $(tcgatables)
```

◼

Which are the “tumor” samples?

C27: ⟪ tumors.query.sql ⟫ ≡  
 root chunk

```
SELECT * FROM sample_type
WHERE "Definition" LIKE '%tumor%';
```

◼

Table 1: `tumors.sql-result`

| Code | Definition | Short Letter Code |
| --- | --- | --- |
| 01 | Primary solid Tumor | TP |
| 02 | Recurrent Solid Tumor | TR |
| 08 | Human Tumor Original Cells | THOC |

Tumor sample types. We are most interested in the type “Primary solid Tumor”.

C28: ⟪ :make Run a query on the relational database ⟫ ≡  
 root chunk

```
%.sql-result: tcga.db sql-result.bash %.query.sql
	bash sql-result.bash $*.query.sql \
	    | sqlite3 tcga.db \
	    > $@
```

◼

A script that runs a query so that the results are returned as an HTML table with headers.

C29: ⟪ sql-result.bash ⟫ ≡  
 root chunk

```
echo '.mode html'
echo '.headers on'
cat "$1"
```

◼

All that is left is to link the barcodes used in the two data matrices to these tables. To do that, we use two tables that have the complete barcode as a unique identifier, and the fields of the barcode as the rest of the columns.

The amplification matrix uses “Patient” barcodes as column labels.

C30: ⟪ ampl.colnames.awk ⟫ ≡  
 root chunk

```
BEGIN {
    FS = "-"; OFS = "\t"
    print "Barcode", "TSS Code", "Patient"
}
{ print $0, $2, $3 }
```

◼

The transcript level matrix uses “Analyte” barcodes as column labels.

C31: ⟪ mrna.colnames.awk ⟫ ≡  
 root chunk

```
BEGIN {
    FS = "-"; OFS = "\t"
    print "Barcode", "TSS Code", "Patient",
          "Sample", "Vial", "Portion", "Analyte"
}
{
    sample_type = substr($4, 1, 2);
    vial = substr($4, 3, 1);
    portion = substr($5, 1, 2);
    analyte = substr($5, 3, 1);
    print $0, $2, $3,
          sample_type, vial, portion, analyte;
}
```

◼

C32: ⟪ :make Matrix column labels to files ⟫ ≡  
 root chunk

```
%_cols.tsv: %.colnames %.colnames.awk
	< $*.colnames ⟪ Tab-separated fields to lines ⟫  \
	    | awk -f $*.colnames.awk \
	    > $@
```

◼

C33: ⟪ Tab-separated fields to lines ⟫ ≡  
 Appears in C32, C61, C65, C87

```
tr "\t" "\n"
```

◼

C34: ⟪ :make Matrix column label files to the database ⟫ ≡  
 root chunk

```
tcga.db: ampl_cols.create-table.sql mrna_cols.create-table.sql
```

◼

What kinds of samples are there in the transcript level matrix?

C35: ⟪ samples.query.sql ⟫ ≡  
 root chunk

```
SELECT
  count("Sample") as n,
  "Definition"
FROM mrna_cols
  INNER JOIN sample_type
    ON mrna_cols."Sample" = sample_type."Code"
GROUP BY "Definition"
ORDER BY n DESC;
```

◼

Table 2: `samples.sql-result`

| n | Definition |
| --- | --- |
| 524 | Primary solid Tumor |
| 59 | Solid Tissue Normal |

Sample types in transcript levels matrix. Most of the samples are from tumors, but some have associated normal tissue controls.

C36: ⟪ patients-in-mrna.query.sql ⟫ ≡  
 root chunk

```
SELECT count(*) as "Number of patients" FROM (
  SELECT DISTINCT "TSS Code", "Patient"
  FROM mrna_cols
);
```

◼

Table 3: `patients-in-mrna.sql-result`

| Number of patients |
| --- |
| 526 |

Number of unique patients.

C37: ⟪ patients-with-control.query.sql ⟫ ≡  
 root chunk

```
SELECT count(*) AS n FROM (
  SELECT x."TSS Code", x."Patient"
  FROM mrna_cols AS x
    INNER JOIN mrna_cols AS y
      ON x."TSS Code" = y."TSS Code"
        AND x."Patient" = y."Patient"
        AND x."Sample" < y."Sample"
);
```

◼

Table 4: `patients-with-control.sql-result`

| n |
| --- |
| 57 |

Number of patients with control sample. This counts all patients that have both tumor and normal solid tissue sample in the transcript level matrix.

How many are the patients from the amplification matrix for which we have tumor samples in the transcript level matrix?

C38: ⟪ patients-tumors.query.sql ⟫ ≡  
 root chunk

```
SELECT count(*) AS 'Cancer patient count'
FROM ( ⟪ ampl-mrna.fragment.sql ⟫ );
```

◼

This SQL query fragment creates a with two columns. In the `ampl` column will be the patient barcodes, and in the `mrna` column the solid tumor sample barcodes of the same patients.

C39: ⟪ ampl-mrna.fragment.sql ⟫ ≡  
 Appears in C38, C48, C72, C73

```
SELECT
  ampl_cols."Barcode" AS ampl,
  mrna_cols."Barcode" AS mrna
FROM ampl_cols
  INNER JOIN mrna_cols
    USING ("TSS Code", "Patient")
  INNER JOIN sample_type
    ON mrna_cols."Sample" = sample_type."Code"
WHERE sample_type."Definition" = 'Primary solid Tumor'
```

◼

Table 5: `patients-tumors.sql-result`

| Cancer patient count |
| --- |
| 516 |

Useful number of patients. These are the patients that have an amplification status, and a transcript level from a solid tumor sample.

Finally, for convenience, we add the Ensembl ID to Gene name mapping downloaded from Ensembl to the database, too. Even though we are able to query this file with the help of Awk and Bash, as demonstrated by ⟪ sanity-check.bash ⟫ and ⟪ ID from name .awk ⟫, it is unnecessarily hacky.

C40: ⟪ :make Ensembl IDs and names to the database ⟫ ≡  
 root chunk

```
tcga.db: ensembl/id_descr_name.create-table.sql
```

◼

Now, to get the Ensembl IDs of genes, it is enough to say:

C41: ⟪ erbb2-stard3-ids.query.sql ⟫ ≡  
 root chunk

```
SELECT
  "Associated Gene Name",
  "Ensembl Gene ID"
FROM id_descr_name
WHERE "Associated Gene Name" IN ( 'ERBB2', 'STARD3' );
```

◼

Table 6: `erbb2-stard3-ids.sql-result`

| Associated Gene Name | Ensembl Gene ID |
| --- | --- |
| STARD3 | ENSG00000131748 |
| ERBB2 | ENSG00000141736 |

Ensembl IDs of *ERBB2* and *STARD3*.

Note that the queries above double as an informal validation for the database.

# Gene association

We will try to find a correlation of the amplification status or the transcript levels between pairs of this set of genes:

C42: ⟪ Genes of interest ⟫ ≡  
 Appears in C43, C44

```
"STARD3", "ERBB2", "NDRG1", "LAPTM4B", "LAPTM4A"
```

◼

## Pairs of genes

To generate the pairs of genes, we could say:

C43: ⟪ :dummy Generate pairs ⟫ ≡  
 root chunk

```
list = { ⟪ Genes of interest ⟫ };
for (i = 0; i < length(list); ++i)
    for (j = i+1; j < length(list); ++j)
        compare(list[i], list[j]);
```

◼

However, we can achieve the same, and more, with SQL:

C44: ⟪ genes-of-interest-pairs.query.sql ⟫ ≡  
 root chunk

```
SELECT DISTINCT
  x."Associated Gene Name" AS name1,
  y."Associated Gene Name" AS name2,
  x."Ensembl Gene ID" AS id1,
  y."Ensembl Gene ID" AS id2
FROM id_descr_name AS x, id_descr_name AS y
WHERE name1 IN ( ⟪ Genes of interest ⟫ )
  AND name2 IN ( ⟪ Genes of interest ⟫ )
  AND name1 < name2
ORDER BY name1;
```

◼

This implicit rule keeps the headers, so the resulting `*.tsv-result` file can be loaded from R using `read.delim` with the default parameters.

C45: ⟪ :make Results of a query to a file ⟫ ≡  
 root chunk

```
%.tsv-result: tcga.db sql-tsv.bash %.query.sql
	bash sql-tsv.bash $*.query.sql \
	    | sqlite3 tcga.db \
	    > $@
```

◼

This is the bash script that sets the mode and turns on the headers.

C46: ⟪ sql-tsv.bash ⟫ ≡  
 root chunk

```
echo '.mode tabs'
echo '.headers on'
cat "$1"
```

◼

## Association results

Now, using this, we can read this file from R and use it to extract the relevant genes (rows) from the amplification status and transcript level matrices.

C47: ⟪ :make ⟫ ≡  
 ⇧ C22

```
genes-of-interest.Rsave: genes-of-interest.R \
                         genes-of-interest-pairs.tsv-result \
                         columns-of-interest.tsv-result \
                         input-data.Rsave
	Rscript --vanilla $^ $@

fig.genes-of-interest.%: plot-genes-of-interest.R \
                         genes-of-interest.Rsave
	Rscript --vanilla $^ $@ $*
```

⇩ C60

C48: ⟪ columns-of-interest.query.sql ⟫ ≡  
 root chunk

```
⟪ ampl-mrna.fragment.sql ⟫;
```

◼

To summarize some of the useful information in this subset of the data, we perform a *χ*2 test on the amplification status from both genes, and a Pearson’s correlation test on the transcript levels from both genes.

C49: ⟪ genes-of-interest.R ⟫ ≡  
 root chunk

```
args.names <- c("from.file", "cols.file", "save.file", "result.file")
⟪ Read and name R command line arguments ⟫
⟪ Load genes of interest data from files ⟫
⟪ Keep only the overlapping columns ⟫

correlation <- function(x) {
    n1 <- x[1]
    n2 <- x[2]
    a1 <- ampl[x[3],]
    a2 <- ampl[x[4],]
    t1 <- mrna[x[3],]
    t2 <- mrna[x[4],]

    chisq.test(a1, a2) -> chisq.r

    ⟪ Color mRNA scatter plot R function ⟫
    scatter.plot.col(a1, a2) -> mrna.colors
    cor.test(t1, t2, method="pearson") -> cor.r

    list(name.a=n1, name.b=n2,
         chisq.result=chisq.r,
         transcript.a=t1, transcript.b=t2,
         mrna.col=mrna.colors,
         pearson.estimate=cor.r$estimate)
}

apply(pairs, 1, correlation) -> result
save(result, file=args["result.file"])
```

◼

Result 1: `fig.genes-of-interest.pdf`

Figure 2: `fig.genes-of-interest.svg`

The results for all possible pairs. The first two plots, a positive control. *STARD3* and *ERBB2* are on the same amplicon and are known to be co-amplified and co-overexpressed in breast canser patient samples.

There is no correlation between *NRDG1* and *STARD3*. They are not co-amplified and the transcript levels do not correlate.

*LAPTM4B* and *STARD3* are not co-amplified. On the transcript level however there is a moderate positive correlation between the two.

*LAPTM4B* is co-amplified with *NDRG1*. This is reflected in the correlation of the transcript levels of the two genes.

C50: ⟪ Load genes of interest data from files ⟫ ≡  
 Appears in C49

```
as.matrix(read.delim(args["from.file"])) -> pairs
as.matrix(read.delim(args["cols.file"])) -> cols
load(args["save.file"])
```

◼

C51: ⟪ Keep only the overlapping columns ⟫ ≡  
 Appears in C49, C94

```
ampl <- ampl[,cols[,"ampl"]]
mrna <- mrna[,cols[,"mrna"]]
```

◼

## Plotting the results

The names are used to label the axes. The transcript levels are used for the scatter plot. The generated colors for the scatter plot were generated above. This uses the list of values to draw summary plots.

C52: ⟪ plot-genes-of-interest.R ⟫ ≡  
 root chunk

```
args.names <- c("result.file", "fig.file", "format")
⟪ Read and name R command line arguments ⟫
load(args["result.file"])

⟪ Open graphics device R function ⟫
r <- length(result)
open.device(args["format"], 
            args["fig.file"],
            width=5.6, height=r*3.8,
            pointsize=14)
⟪ Define layout for correlation plots R function ⟫
cor.plots.layout(r)

⟪ Draw correlation plots R function ⟫
lapply(result, draw.cor.plots) -> foo

dev.off() -> foo
```

◼

Unfortunately, I could not figure out an easy way to choose the graphics device at run-time. Instead, I am using this hack to open the correct device based on a character argument:

C53: ⟪ Open graphics device R function ⟫ ≡  
 Appears in C52, C78, C83, C100

```
open.device <- function(format, ...) {
    if (format == "pdf") {
        pdf(...)
    } else if (format == "svg") {
        svg(...)
    }
}
```

◼

This defines a layout with as many rows as items in the `result` variable, and splits each row in two columns.

C54: ⟪ Define layout for correlation plots R function ⟫ ≡  
 Appears in C52, C100

```
cor.plots.layout <- function(r) {
    layout(matrix(1:(2*r), nrow=r, ncol=2, byrow=T),
           widths=c(4,7),
           heights=rep(1, r))
}
```

◼

When below a limit, display numerical value differently.

C55: ⟪ Generate a subtitle ⟫ ≡  
 Appears in C56

```
make.subtitle.str <- function(str, limit, prec, x) {
    if (x < limit) {
        subtitle.str <- paste(str, "<", limit)
    } else {
        subtitle.str <- paste(str, "=", round(x, prec))
    }
}
```

◼

C56: ⟪ Draw correlation plots R function ⟫ ≡  
 Appears in C52, C100

```
draw.cor.plots <- function(x) {
    ⟪ Generate a subtitle ⟫
    ampl.col <- hcl(c(150,330), l=67)
    ⟪ Draw a barplot to visualize amplification status correlation ⟫
    ⟪ Draw a scatter plot to visualize transcript level correlation ⟫
}
```

◼

This uses the observed 2 × 2 contingency table from the *χ*2 test to plot each value as a bar height.

C57: ⟪ Draw a barplot to visualize amplification status correlation ⟫ ≡  
 Appears in C56

```
subtitle.str <- make.subtitle.str("p-val", 0.005, 3, x$chisq.result$p.value)
cont.table <- t(x$chisq.result$observed)
foo <- c("basal", "amplified")
dimnames(cont.table) <- list(foo, foo)

barplot(cont.table, beside=T,
        col=ampl.col,
        main="Amplification",
        sub=subtitle.str,
        xlab=paste(x$name.a),
        ylab="number of patients")

legend("topright",
       title=paste(x$name.b),
       legend=foo,
       fill=ampl.col, border=ampl.col,
       bty="n")
```

◼

C58: ⟪ Draw a scatter plot to visualize transcript level correlation ⟫ ≡  
 Appears in C56

```
subtitle.str <- make.subtitle.str("cor", 0.1, 2, x$pearson.estimate)
plot(x$transcript.a, x$transcript.b,
     col=x$mrna.col,
     main=paste("Transcipt levels"),
     sub=subtitle.str,
     xlab=x$name.a, ylab=x$name.b,
     bty="n")

legend("topleft",
    title = "Amplification",
    legend = c("amplified", "basal", "different"),
    pch = c(1, 1, 1),
    col=c("darkred", "darkblue", "olivedrab"),
    bty = "n")
```

◼

In addition to using the actual transcript levels from the two patients, it also uses the amplification status for each patient to draw the circle in blue (both negative), red (both positive), or green (different). This implementation works because the amplification statuses of both genes are represented as logical vectors, and we know that all `NA`’s have been substituted with `FALSE`. When used in ordinary arithmetic, logical vectors are coerced into numeric vectors, with `FALSE = 0` and `TRUE = 1`.

C59: ⟪ Color mRNA scatter plot R function ⟫ ≡  
 Appears in C49, C100

```
scatter.plot.col <- function(a1, a2) {
    c("darkblue", "olivedrab", "darkred")[1+a1+a2]
}
```

◼

# Survival

The next question is whether there is a correlation between gene amplification and transcription levels and patient survival.

## Clinical patient data

The data on patient survival is extracted from a table with many cliniclal parameters.

S7: `clinical/clinical_patient_all_brca.txt`

Listing 5: `clinical-column-number`

```
102
```

Number of columns. Each column in this matrix is one clinical parameter of that patient. The majority are not interesting at this stage.

C60: ⟪ :make ⟫ ≡  
 ⇧ C47

```
clinical-column-number: clinical/clinical_patient_all_brca.txt
	< $< ⟪ Get first row ⟫ | wc --words > $@
```

⇩ C61

Which could be the relevant rows? Time is likely to be measured in days or months.

Listing 6: `clinical-relevant-columns`

```
24:days_to_birth
25:days_to_death
26:days_to_initial_pathologic_diagnosis
27:days_to_last_followup
28:days_to_last_known_alive
```

Columns that could be relevant. We need the days to death (25), and last followup (27) or last known alivem (28), whichever is longer.

C61: ⟪ :make ⟫ ≡  
 ⇧ C60

```
clinical-relevant-columns: clinical/clinical_patient_all_brca.txt
	< $< ⟪ Get first row ⟫ \
	    | ⟪ Tab-separated fields to lines ⟫ \
	    | grep --line-number 'days\|months' \
	    > $@
```

⇩ C62

Extract the relevant columns only: Patient barcode (1), and days to death (25), to last followup (27), and to last known alive (28).

C62: ⟪ :make ⟫ ≡  
 ⇧ C61

```
patient-days: clinical/clinical_patient_all_brca.txt
	< $^ ⟪ Drop first row ⟫ | cut --fields=1,25,27,28 > $@
```

⇩ C63

Note that column 26, “Days to initial pathologic diagnosis”, has a value of 0 for every one of the 863 patients:

Listing 7: `days-to-initial`

```
    863 0
```

A useless column.

C63: ⟪ :make ⟫ ≡  
 ⇧ C62

```
days-to-initial: clinical/clinical_patient_all_brca.txt
	⟪ Drop first row ⟫ $< | cut --fields=26 | sort | uniq -c > $@
```

⇩ C64

Are there field values that are *not* numbers? Which are they? How many of each?

Listing 8: `patient-days-nans`

```
   1706 [Not Available]
```

Field values that are not numbers. There is only one value that is not a number: the string “[Not Available]”.

C64: ⟪ :make ⟫ ≡  
 ⇧ C63

```
patient-days-nans: nan-fields.sh patient-days
	bash $^ > $@
```

⇩ C75

C65: ⟪ nan-fields.sh ⟫ ≡  
 root chunk

```
⟪ Drop first row ⟫ "$1" \
    | ⟪ Drop first column ⟫ \
    | ⟪ Tab-separated fields to lines ⟫ \
    | sed -n '/^[0-9.]\+$/!p' \
    | sort \
    | uniq --count
```

◼

C66: ⟪ :make Normalized patient survival file ⟫ ≡  
 root chunk

```
patient_survival.rows-tsv: patient_survival.awk patient-days
	awk -f $^ > $@
```

◼

To prepare this table for the database, we normalize it first. This means that there will be one record in the database for each Patient-Value combination. All “[Not Available]” values will be skipped.

C67: ⟪ patient\_survival.awk ⟫ ≡  
 root chunk

```
BEGIN {
    FS = "\t" ; OFS = "\t"
    col[2] = "Death"
    col[3] = "Last followup"
    col[4] = "Last known alive"
}
{
    for (i = 2; i <= 4; ++i) {
        if ($i != "[Not Available]") {
            print $1, col[i], $i
        }
    }
}
```

◼

The normalized table is inserted to the relational database.

C68: ⟪ :make Patient survival to the database ⟫ ≡  
 root chunk

```
tcga.db: patient_survival.define-insert-table.sql
```

◼

Because the last column in the database is a number, it is better to manually define the schema for the table that is going to hold this data.

C69: ⟪ patient\_survival.schema.sql ⟫ ≡  
 root chunk

```
CREATE TABLE patient_survival (
  "Barcode" TEXT,
  "Event" TEXT,
  "Days" INTEGER
);
```

◼

C70: ⟪ :make Implicit rule for SQL tables with an explicit schema ⟫ ≡  
 root chunk

```
%.define-insert-table.sql: define-insert-table.bash %.schema.sql %.rows-tsv
	bash $^ > $@
```

◼

In addition to ⟪ create-table.bash ⟫, this script takes an explicitly defined schema.

C71: ⟪ define-insert-table.bash ⟫ ≡  
 root chunk

```
NAME=$(basename --suffix=.schema.sql "$1")
echo 'DROP TABLE IF EXISTS "'"$NAME"'";'
cat "$1"
echo '.separator "\t"'
echo ".import '$2' $NAME"
```

◼

## Long-term survival

We already know from ⟪ :table patients-tumors.sql-result ⟫ that there are 516 patients for whom we have both amplification and transcript level data. Now we can ask, for how many of them do we have an event (patient dies) within the first 5 years?

C72: ⟪ five-year-events.query.sql ⟫ ≡  
 root chunk

```
WITH ampl_mrna AS ( ⟪ ampl-mrna.fragment.sql ⟫ )
SELECT count(*) AS "Number of events"
FROM patient_survival
  INNER JOIN ampl_mrna
    ON ampl_mrna.ampl = "Barcode"
WHERE "Event" = 'Death'
  AND "Days" < 365*5;
```

◼

Table 7: `five-year-events.sql-result`

| Number of events |
| --- |
| 39 |

Number of events within the first 5 years.

And finally, we need a table that we can use to do the survival statistics in R. For the survival statistics, we need at the very least a table with two columns: one with the follow-up times, and one with the status indicator with 0 for alive and 1 for dead. We also will need the patient barcodes for the amplification dataset and the sample barcode for the transcript level dataset.

C73: ⟪ survival-table.query.sql ⟫ ≡  
 root chunk

```
WITH
  ampl_mrna AS ( ⟪ ampl-mrna.fragment.sql ⟫ ),
  patient_event_time AS (
    SELECT
      "Barcode" AS barcode,
      "Event" AS event,
      MAX("Days"/365.25) AS years
    FROM patient_survival
    GROUP BY barcode
  )
SELECT
  ampl AS ampl_patient,
  mrna AS mrna_sample,
  CASE
    WHEN event = 'Death' AND years <= 5 THEN 1
    WHEN event <> 'Death' OR years > 5 THEN 0
  END AS status,
  CASE
    WHEN years <= 5 THEN years
    ELSE 5
  END AS time
FROM patient_event_time
  INNER JOIN ampl_mrna ON ampl_patient = barcode;
```

◼

And, of course, the IDs of the genes we are interested in:

C74: ⟪ survival-genes.query.sql ⟫ ≡  
 root chunk

```
SELECT
  "Associated Gene Name" AS name,
  "Ensembl Gene ID" AS id
FROM id_descr_name
WHERE name IN ('STARD3', 'LAPTM4B', 'NDRG1', 'SERPINA1');
```

◼

In order to avoid loading the complete data matrices multiple times, we will load them once and save only the four genes of interest, and only the columns we need.

C75: ⟪ :make ⟫ ≡  
 ⇧ C64

```
survival-genes.Rsave: survival-genes.R \
                      input-data.Rsave \
                      survival-table.tsv-result \
                      survival-genes.tsv-result
	Rscript --vanilla $^ $@
```

⇩ C77

C76: ⟪ survival-genes.R ⟫ ≡  
 root chunk

```
args.names <- c("input.file",
                "clinical.file",
                "genes.file",
                "save.file")
⟪ Read and name R command line arguments ⟫

read.delim(args["clinical.file"]) -> clinical
attach(clinical)
as.character(ampl_patient) -> ampl.patient
as.character(mrna_sample) -> mrna.sample

load(args["input.file"])
ampl[,ampl.patient] -> ampl
mrna[,mrna.sample] -> mrna

as.matrix(read.delim(args["genes.file"])) -> genes

ampl.survival <- ampl[genes[,"id"],]
rownames(ampl.survival) <- genes[,"name"]
mrna.survival <- mrna[genes[,"id"],]
rownames(mrna.survival) <- genes[,"name"]

save(status, time, ampl.survival, mrna.survival, file=args["save.file"])
```

◼

Result 2: `survival-genes.Rsave`

## Effects on long-term patient survival

Now we determine the correlation between the amplification status, transcipt levels, and patient survival. First, we split patients in two groups. For the amplification data, the two groups are “basal” and “amplified”. For the transcript level data, the two groups are simply levels below and above the median within this gene.

C77: ⟪ :make ⟫ ≡  
 ⇧ C75

```
%-survival-curve.svg: survival-curve.R survival-genes.Rsave
	Rscript --vanilla $^ $* $@ svg

%-survival-curve.pdf: survival-curve.R survival-genes.Rsave
	Rscript --vanilla $^ $* $@ pdf
```

⇩ C82

Figure 3: `SERPINA1-survival-curve.svg`

Survival curve for *SERPINA1*. *SERPINA1* amplification status and transcript levels are positively correlated with patient survival, as reported previously.

Figure 4: `STARD3-survival-curve.svg`

Survival curve for *STARD3*.

Figure 5: `NDRG1-survival-curve.svg`

Survival curve for *NDRG1*. *NDRG1* amplification and upregulation of transcript levels both have a weak negative effect on breast cancer patient survival.

Figure 6: `LAPTM4B-survival-curve.svg`

Survival curve for *LAPTM4B*. *LAPTM4B* amplification has a statistically significant effect on the survival of breast cancer patients. Transcript levels upregulation exhibits a similar tendency, but the difference is not statistically significant.

Result 3: `SERPINA1-survival-curve.pdf`

Result 4: `NDRG1-survival-curve.pdf`

Result 5: `LAPTM4B-survival-curve.pdf`

C78: ⟪ survival-curve.R ⟫ ≡  
 root chunk

```
args.names <- c("save.file", "gene.name", "fig.file", "format")
⟪ Read and name R command line arguments ⟫
⟪ Load survival saved state ⟫
⟪ Survival statistics ⟫

⟪ Open graphics device R function ⟫
open.device(args["format"],
            args["fig.file"], width=8, height=4.4, pointsize=11)
par(mfrow=c(1,2))
⟪ Plot survival R function ⟫
plot.survival(list(fit=survfit.ampl, diff=survdiff.ampl),
              main.text="Amplification",
              title.text=paste(gn, "amplification"),
              legend.text=c("basal", "amplified"))
plot.survival(list(fit=survfit.mrna, diff=survdiff.mrna),
              main.text="Transcript levels",
              title.text=paste(gn, "transcript"),
              legend.text=c("below median", "above median"))
dev.off() -> foo
```

◼

C79: ⟪ Load survival saved state ⟫ ≡  
 Appears in C78

```
load(args["save.file"])
args["gene.name"] -> gn
ampl.survival[gn,] -> ampl
mrna.survival[gn,] -> mrna
```

◼

The survival statistics are done with the help of `library(survival)`.

C80: ⟪ Survival statistics ⟫ ≡  
 Appears in C78

```
mrna > median(mrna) -> mrna.high

library(survival)

Surv(time, status) -> so
survfit(so ~ ampl) -> survfit.ampl
survdiff(so ~ ampl) -> survdiff.ampl
survfit(so ~ mrna > median(mrna, na.rm=T)) -> survfit.mrna
survdiff(so ~ mrna > median(mrna, na.rm=T)) -> survdiff.mrna
```

◼

The p-value is calculated from the *χ*2 test statistic with one degree of freedom (this is always the case, since we always have two groups, and both have patients in them). A table with the “numbers at risk” is added to directly to the plot.

C81: ⟪ Plot survival R function ⟫ ≡  
 Appears in C78, C83

```
plot.survival <- function(x, main.text, title.text, legend.text) {
    pval <- 1 - pchisq(x$diff$chisq, 1)
    cols <- c("darkblue", "darkred")
    plot(x$fit,
         main=main.text,
         ylab="Survival", xlab="Years",
         xlim=c(-0.4,5.3), ylim=c(0.5,1),
         col=cols,
         bty="n")
    legend("bottomleft",
           legend=legend.text,
           lty=1,
           bty="n",
           col=cols)
    legend("bottomright",
           bty="n",
           legend=paste("p=", signif(pval, digits=2), sep=""))
    text(x=-0.3, y=0.71, labels="Numbers at risk", pos=4, offset=0)
    text(x=0:5,
         y=rep(c(0.67,0.63), each=6),
         col=rep(cols, each=6),
         labels=summary(x$fit, times=0:5)$n.risk)
}
```

◼

Is there an additive (negative) effect of *LAPTM4B* and *NDRG1* on patient survival?

C82: ⟪ :make ⟫ ≡  
 ⇧ C77

```
%-survival-curve-combined.svg: survival-curve-combined.R \
                               survival-genes.Rsave
	Rscript --vanilla $^ $* $@ svg

%-survival-curve-combined.pdf: survival-curve-combined.R \
                               survival-genes.Rsave
	Rscript --vanilla $^ $* $@ pdf
```

⇩ C87

Figure 7: `NDRG1-LAPTM4B-survival-curve-combined.svg`

Combined effect of *NDRG1* and *LAPTM4B* on survival. On the left hand side the patients are separated in two groups: in the group “both high” (in red), the transcript levels for both patients are above median. In the other group, “different” (in blue), are all other patients. On the right hand side, patients with different mRNA expression levels for the two trancripts were removed from the data set. In the “high” group (in red) are the patients with both transcript levels high, while in the “low” group (in blue) are the patients that have both transcript levels low.

Result 6: `NDRG1-LAPTM4B-survival-curve-combined.pdf`

C83: ⟪ survival-curve-combined.R ⟫ ≡  
 root chunk

```
args.names <- c("save.file", "gene1.gene2.names", "fig.file", "format")
⟪ Read and name R command line arguments ⟫
⟪ Names and transcript levels of the two genes ⟫
apply(mrna, 1, median, na.rm=T) -> mrna.median
mrna > mrna.median -> is.high
apply(is.high, 2, all) -> both.high

library(survival)
⟪ Patient survival: both high vs. different ⟫ -> high.different
⟪ Patient survival: both high vs. both low ⟫ -> high.low

⟪ Open graphics device R function ⟫
open.device(args["format"],
            args["fig.file"], width=8, height=4.4, pointsize=11)
par(mfrow=c(1,2))
⟪ Plot survival R function ⟫
plot.survival(high.different,
              main.text="Different vs. High",
              legend.text=c("different", "both high"))
plot.survival(high.low,
              main.text="Low vs. High",
              legend.text=c("both low", "both high"))
dev.off() -> foo
```

◼

C84: ⟪ Names and transcript levels of the two genes ⟫ ≡  
 Appears in C83

```
load(args["save.file"])
strsplit(args["gene1.gene2.names"],
         split="-", fixed=T)[[1]] -> gene.names
mrna.survival[gene.names,] -> mrna
```

◼

C85: ⟪ Patient survival: both high vs. different ⟫ ≡  
 Appears in C83

```
Surv(time, status) -> so
list(fit=survfit(so ~ both.high), diff=survdiff(so ~ both.high))
```

◼

C86: ⟪ Patient survival: both high vs. both low ⟫ ≡  
 Appears in C83

```
mrna < mrna.median -> is.low
apply(is.low, 2, all) -> both.low
both.high | both.low -> filtered
time[filtered] -> time.f
status[filtered] -> status.f
both.high[filtered] -> both.high.f

Surv(time.f, status.f) -> so.f
list(fit=survfit(so.f ~ both.high.f), diff=survdiff(so.f ~ both.high.f))
```

◼

# Protein complexes regulated by micro-RNA

Is there a correlation between the predicted targets of the same miRNA? This question is motivated by the known phenomenon of miRNA targetting a complex of functionally related proteins. The predicted targets are collected using a short-list of experimentally validated micro-RNAs. Then, these predicted targets for each gene are correlated to the gene using the available mRNA levels from patient solid tumors.

## Micro-RNAs and predicted targets

To answer this question, we will try to find experimentally validated micro-RNAs that target the genes of interest, as listed in ⟪ Genes of interest ⟫. We drop *STARD3* from that list, as it correlates very strongly with *ERBB2* and is far less studied experimentally; we also drop *LAPTM4A*, as it showed no correlation to any of the other genes, and is also poorly studied. We are left with *ERBB2*, *NDRG1*, and *LAPTM4B*. The main criteria for choosing a miRNA that targets each gene is that it downregulates the gene in the context of cancer progression.

For *ERBB2*, hsa-miR-155-5p (MIMAT0000646) was shown to downregulate ErbB2 and suppress ErbB2-induced malignant transformation of breast epithelial cells by two distinct mechanism: repressing ErbB2 transcription, and directly targetting ErbB2 via a regulatory element (doi:10.1038/onc.2016.132).

For *NDRG1*, miR-769-3p (MIMAT0003887) was found to down-regulate *NDRG1* in MCF-7 cells during reoxygenation (doi:10.1038/srep05908).

For *LAPTM4B*, miR-188-5p (MIMAT0000457) was found to inhibit tumor growth and metastasis by repressing *LAPTM4B* expression, acting as a tumor supressor (doi:10.18632/oncotarget.3341).

Using miRWalk2.0:

- Predicted Target Module → MicroRNA-Gene Targets
- Human, miRBase, MIMATid
- Identifiers:

```
MIMAT0000646
MIMAT0003887
MIMAT0000457
```

- Other databases: miRWalk, miRanda, miRDB, RNA22, Targetscan
- “Putative target genes predicted by chosen algorithms within mRNA selected regions” → 3UTR

The downloaded file:

S8: `microrna/3utr-comparative.tsv`

What are the columns in this file?

Listing 9: `microrna/table-header`

```
     1	miRNA
     2	MIMATid
     3	Gene
     4	EntrezID
     5	RefseqID
     6	miRWalk
     7	miRanda
     8	miRDB
     9	RNA22
    10	Targetscan
    11	SUM
```

Column labels.

C87: ⟪ :make ⟫ ≡  
 ⇧ C82

```
microrna/table-header: microrna/3utr-comparative.tsv
	⟪ Get first row ⟫ $^ \
	    | ⟪ Tab-separated fields to lines ⟫ \
	    | nl \
	    > $@
```

⇩ C89

For now, we are interested in the miRNA identifier (1), the predicted target gene (3), the IDs of the mRNA sequences being targetted, and the number of positive predictions for that gene, commonly used as an indicator of the prediction strength (11). We will a table with these columns to the relational database.

C88: ⟪ mirna.schema.sql ⟫ ≡  
 root chunk

```
CREATE TABLE mirna (
  mirna TEXT,
  gene TEXT,
  refseqid TEXT,
  sum INTEGER
);
```

◼

C89: ⟪ :make ⟫ ≡  
 ⇧ C87

```
tcga.db: mirna.define-insert-table.sql
mirna.rows-tsv: microrna/3utr-comparative.tsv
	⟪ Drop first row ⟫ $^ \
	    | cut --fields=1,3,5,11 \
	    > $@
```

⇩ C90

To be able to find the intersection of genes that are in the predicted targets, *and* in the transcript level matrix, we will insert the row labels of the two matrices in the relational database.

C90: ⟪ :make ⟫ ≡  
 ⇧ C89

```
tcga.db: ampl_rownames.create-table.sql \
         mrna_rownames.create-table.sql
%_rownames.tsv: %.rownames
	echo "Ensembl Gene ID" > $@
	cat $^ >> $@
```

⇩ C97

To be able to caclulate the transcript level correlation for each of these genes, we need the Ensembl Gene IDs for the predictions for each of the three miRNAs. Note the reuse of `columns-of-interest.tsv-result`, generated in ⟪ columns-of-interest.query.sql ⟫.

C91: ⟪ :make Calculate and save the mRNA correlation coefficients ⟫ ≡  
 root chunk

```
mirna-mrna.Rsave: mirna-mrna.R \
                  input-data.Rsave \
                  columns-of-interest.tsv-result \
                  mirna-genes.tsv-result \
                  hsa-miR-155-5p.mirna-predictions.tsv \
                  hsa-miR-188-5p.mirna-predictions.tsv \
                  hsa-miR-769-3p.mirna-predictions.tsv
	Rscript --vanilla $^ $@

mirna-counts.txt: hsa-miR-155-5p.mirna-predictions.tsv \
                  hsa-miR-188-5p.mirna-predictions.tsv \
                  hsa-miR-769-3p.mirna-predictions.tsv
	wc --lines $^ > $@

%.mirna-predictions.tsv: mirna-predictions.bash tcga.db
	bash $^ $* > $@
```

◼

Listing 10: `mirna-counts.txt`

```
  957 hsa-miR-155-5p.mirna-predictions.tsv
  826 hsa-miR-188-5p.mirna-predictions.tsv
  770 hsa-miR-769-3p.mirna-predictions.tsv
 2553 total
```

Number of targets for each gene.

C92: ⟪ mirna-genes.query.sql ⟫ ≡  
 root chunk

```
WITH gene_mirna ("Associated Gene Name", mirna) AS (
  VALUES
    ('ERBB2', 'hsa-miR-155-5p'),
    ('NDRG1', 'hsa-miR-769-3p'),
    ('LAPTM4B', 'hsa-miR-188-5p')
)
SELECT
  "Associated Gene Name" AS name,
  "Ensembl Gene ID" AS id,
  mirna
FROM id_descr_name
  NATURAL INNER JOIN gene_mirna;
```

◼

Result 7: `mirna-genes.tsv-result`

C93: ⟪ mirna-predictions.bash ⟫ ≡  
 root chunk

```
sqlite3 "$1" << end_of_file
.header on
.separator "\t"
WITH gene_count_sum AS (
  SELECT
    gene,
    count(refseqid) AS count,
    sum
  FROM mirna
  WHERE mirna = '$2'
  GROUP BY gene, sum
)
SELECT DISTINCT
  gene,
  "Ensembl Gene ID" AS id
FROM gene_count_sum
  INNER JOIN id_descr_name
    ON gene = "Associated Gene Name"
  NATURAL INNER JOIN ampl_rownames
  NATURAL INNER JOIN mrna_rownames
WHERE sum >= 4
  OR (sum = 3 AND count >= 2);
end_of_file
```

◼

C94: ⟪ mirna-mrna.R ⟫ ≡  
 root chunk

```
args.names <- c("input",
                "cols",
                "genes",
                "one",
                "two",
                "three",
                "result")
⟪ Read and name R command line arguments ⟫

as.matrix(read.delim(args["genes"])) -> mirna.gene.id
mirna.gene.id[,c("name","id")] -> gene.id
mirna.gene.id[,"mirna"] -> rownames(gene.id)

⟪ Load miRNA targets R function ⟫
lapply(list("one", "two", "three"),
       load.mirna.targets) -> mirna.targets
lapply(mirna.targets,
       function(x) { x$gene["name"] }) -> names(mirna.targets)

load(args["input"])
as.matrix(read.delim(args["cols"])) -> cols
⟪ Keep only the overlapping columns ⟫

pearson.cor <- function(x, y) {
    cor.test(x, y, method="pearson")$estimate
}

⟪ Shortlist highly correlated targets R function ⟫
lapply(mirna.targets, target.cor) -> gene.complex

save(gene.complex, file=args["result"])
```

◼

C95: ⟪ Load miRNA targets R function ⟫ ≡  
 Appears in C94

```
load.mirna.targets <- function(arg.name) {
    strsplit(args[arg.name],
             split=".", fixed=T)[[1]][1] -> mirna.name
    as.matrix(read.delim(args[arg.name])) -> mirna.targets
    mirna.targets[,"id"] -> target.ids
    mirna.targets[,"gene"] -> names(target.ids)
    list(mirna=mirna.name,
         gene=gene.id[mirna.name,],
         targets=target.ids)
}
```

◼

C96: ⟪ Shortlist highly correlated targets R function ⟫ ≡  
 Appears in C94

```
target.cor <- function(x) {
    mrna[x$gene["id"],] -> gene.mrna
    mrna[x$targets,] -> targets.mrna
    names(x$targets) -> rownames(targets.mrna)
    apply(targets.mrna, 1, pearson.cor, gene.mrna) -> target.cors
    sort(target.cors[target.cors > 0.33], decreasing=T) -> shortlist
    targets.mrna[names(shortlist),] -> targets.mrna
    ampl[x$gene["id"],] -> gene.ampl
    ampl[x$targets,] -> targets.ampl
    names(x$targets) -> rownames(targets.ampl)
    targets.ampl[names(shortlist),] -> targets.ampl
    
    list(mirna=x$mirna,
         hits=shortlist,
         gene.ampl=gene.ampl,
         ampl=targets.ampl,
         gene.mrna=gene.mrna,
         mrna=targets.mrna)
}
```

◼

Listing 11: `mirna-mrna-result.txt`

```
Gene:  ERBB2 
miRNA:  hsa-miR-155-5p 
Targets:
[1] "RAB27B" "GPD1L" 

Gene:  LAPTM4B 
miRNA:  hsa-miR-188-5p 
Targets:
 [1] "LAPTM4B"  "YWHAZ"    "JMJD6"   
 [4] "SLMO2"    "TXNRD1"   "C1orf106"
 [7] "RBL1"     "CDC25B"   "PVR"     
[10] "TMEM194A" "PCMT1"    "UQCRB"   
[13] "UCK2"     "FBXO45"   "OSGIN2"  
[16] "USP31"    "SNX22"    "AQP9"    
[19] "SPAST"   

Gene:  NDRG1 
miRNA:  hsa-miR-769-3p 
Targets:
 [1] "NDRG1"    "C11orf86" "SLC6A2"  
 [4] "KIF1B"    "TRIM2"    "CDCA8"   
 [7] "STAC"     "HIC2"     "FOXK2"   
[10] "KLF11"    "ASAP1"    "PHKG1"   
[13] "MFGE8"    "RBM38"
```

The predicted targets with high correlation on the transcript level.

C97: ⟪ :make ⟫ ≡  
 ⇧ C90

```
mirna-mrna-result.txt: mirna-mrna-result.txt.R mirna-mrna.Rsave
	Rscript --vanilla $^ $@
```

◼

C98: ⟪ mirna-mrna-result.txt.R ⟫ ≡  
 root chunk

```
args.names <- c("data", "result")
⟪ Read and name R command line arguments ⟫
load(args["data"])

print.gene.complex <- function(i) {
    cat("Gene: ", names(gene.complex)[[i]], "\n")
    cat("miRNA: ", gene.complex[[i]]$mirna, "\n")
    cat("Targets:\n")
    print(names(gene.complex[[i]]$hits))
    cat("\n")
}

sink(file=args["result"])
options(width=40)
lapply(seq_along(gene.complex), print.gene.complex) -> foo
sink(file=NULL)
```

◼

Which are the interesting hits in this shortlist?

For *LAPTM4B*, there are several interesting hits. One is *PVR*, encoding the Poliovirus receptor protein, which mediates natural killer (NK) cell adhesion and triggers NK cell effector functions. It might provide tumors with a mechanism of immunoevasion, and it plays a role in mediating tumor cell invasion and migration.

Another interesting gene is *SNX22*, encoding the Sorting nexin-22 protein, which may be involved in several stages of intracellular trafficking (By similarity). It interacts with membranes containing phosphatidylinositol 3-phosphate (PtdIns(3P)).

Finally, *SPAST* (Spastin) is a protein that is required for the membrane traffic from the ER to the Golgi.

Now, visualize the amplification status and transcript level correlation between *LAPTM4B* and each of these three genes, as available in the patient solid tumor samples.

C99: ⟪ :make Draw correlation plots for miRNA targets ⟫ ≡  
 root chunk

```
%-mirna.svg: plot-mirna-target.R mirna-mrna.Rsave
	Rscript --vanilla $^ $@ $* svg

%-mirna.pdf: plot-mirna-target.R mirna-mrna.Rsave
	Rscript --vanilla $^ $@ $* pdf
```

◼

Result 8: `LAPTM4B-PVR-mirna.pdf`

Result 9: `LAPTM4B-SNX22-mirna.pdf`

Result 10: `LAPTM4B-SPAST-mirna.pdf`

Figure 8: `LAPTM4B-PVR-mirna.svg`

*PVR*.

Figure 9: `LAPTM4B-SNX22-mirna.svg`

*SNX22*.

Figure 10: `LAPTM4B-SPAST-mirna.svg`

*SPAST*.

C100: ⟪ plot-mirna-target.R ⟫ ≡  
 root chunk

```
args.names <- c("data", "plot", "genes", "format")
⟪ Read and name R command line arguments ⟫
load(args["data"])
strsplit(args["genes"], split="-", fixed=T)[[1]] -> gene.names
gene.names[1] -> gene.of.interest
gene.names[2] -> mirna.target
gene.complex[[gene.of.interest]] -> g
a1 <- g$gene.ampl
a2 <- g$ampl[mirna.target,]

⟪ Color mRNA scatter plot R function ⟫
col <- scatter.plot.col(a1, a2)

⟪ Open graphics device R function ⟫
open.device(args["format"], args["plot"],
            width=7.5, height=5.2, pointsize=12)

⟪ Define layout for correlation plots R function ⟫
cor.plots.layout(1)

⟪ Draw correlation plots R function ⟫

draw.cor.plots(list(name.a=gene.of.interest,
                    name.b=mirna.target,
                    chisq.result=chisq.test(a1, a2),
                    transcript.a=g$gene.mrna,
                    transcript.b=g$mrna[mirna.target,],
                    mrna.col=col,
                    pearson.estimate=g$hits[mirna.target]))

dev.off() -> foo
```

◼
